# To Fix or Not to Fix: Maintenance of Chromosome Ends Versus Repair of DNA Double-Strand Breaks

**DOI:** 10.3390/cells11203224

**Published:** 2022-10-14

**Authors:** Erika Casari, Marco Gnugnoli, Carlo Rinaldi, Paolo Pizzul, Chiara Vittoria Colombo, Diego Bonetti, Maria Pia Longhese

**Affiliations:** Dipartimento di Biotecnologie e Bioscienze, Università degli Studi di Milano–Bicocca, 20126 Milano, Italy

**Keywords:** telomere, double-strand breaks, checkpoint, senescence, *S. cerevisiae*, cancer

## Abstract

Early work by Muller and McClintock discovered that the physical ends of linear chromosomes, named telomeres, possess an inherent ability to escape unwarranted fusions. Since then, extensive research has shown that this special feature relies on specialized proteins and structural properties that confer identity to the chromosome ends, thus allowing cells to distinguish them from intrachromosomal DNA double-strand breaks. Due to the inability of conventional DNA replication to fully replicate the chromosome ends and the downregulation of telomerase in most somatic human tissues, telomeres shorten as cells divide and lose this protective capacity. Telomere attrition causes the activation of the DNA damage checkpoint that leads to a cell-cycle arrest and the entering of cells into a nondividing state, called replicative senescence, that acts as a barrier against tumorigenesis. However, downregulation of the checkpoint overcomes this barrier and leads to further genomic instability that, if coupled with re-stabilization of telomeres, can drive tumorigenesis. This review focuses on the key experiments that have been performed in the model organism *Saccharomyces cerevisiae* to uncover the mechanisms that protect the chromosome ends from eliciting a DNA damage response, the conservation of these pathways in mammals, as well as the consequences of their loss in human cancer.

## 1. History of the Discovery of Telomeres and Telomerase

**Figure 1:**
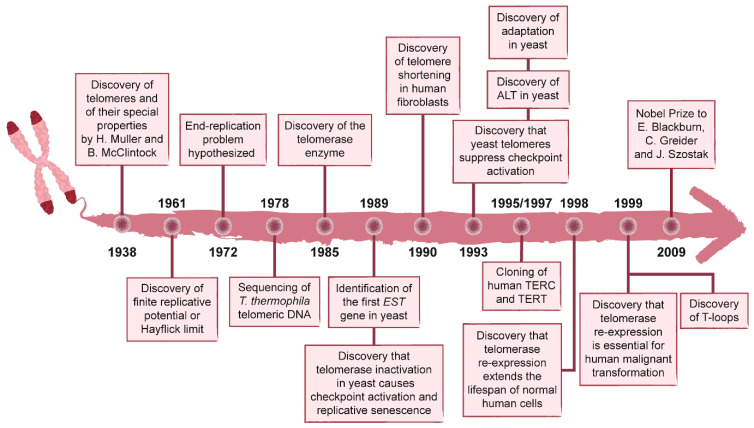
Timeline of the major discoveries in the telomere field.

The concept of telomere was born in the first half of the twentieth century, when Hermann J. Muller, working with the fruit fly *Drosophila melanogaster*, found that X-rays could generate broken chromosomes that can fuse to each other leading to inversions, deletions, and/or translocations (Figure 1). However, these structural alterations never involved the chromosome termini. This finding led him to propose the existence of a “terminal gene” that “must have a special function, that of sealing the ends of the chromosome, so to speak, and that for some reason, a chromosome cannot persist indefinitely without having its ends thus sealed” [1]. Muller called this gene “telomere”, from the Greek *telos* “end” and *meros* “part”.

The idea that the telomere conferred identity to the natural ends of a chromosome, such that a cell could distinguish them from the ends of intrachromosomal double-strand breaks (DSBs), was confirmed soon thereafter by Barbara McClintock, during her cytological studies on irradiated maize chromosomes. She found that a broken chromosome frequently fused with another broken end to produce a dicentric chromosome that would break at the next mitosis, when the two centromeres are pulled apart toward opposite poles of the mitotic spindle. Such broken ends can fuse with other broken ends, starting the so-called “breakage-fusion-bridge cycle” [2]. However, she did not detect fusions involving telomeres, suggesting that one function of the telomere was to protect the natural chromosome ends from fusing to each other.

Following the discovery that telomeres should have special properties, a critical experiment to study telomere function was performed in *Saccharomyces cerevisiae* by Sandell and Zakian, who placed the recognition site of the HO endonuclease 20 kb away from the dispensable left telomere of chromosome VII, such that the telomere can be lost upon HO expression [3]. After elimination of the telomeric DNA, they found that cells underwent an arrest of cell-cycle progression due to activation of the DNA damage checkpoint, indicating that telomeres were essential to prevent the natural chromosome ends from being recognized as DSBs by the checkpoint machinery.

The sequence of telomeric DNA was first identified by Blackburn and Gall in the ciliated protozoan *Tetrahymena thermophila*. They found that the ends of macronuclear ribosomal DNA (rDNA) molecules in this organism consisted of a variable number of 5′-CCCCAA-3′ repeats [4]. Sequencing of the telomeric DNA from other eukaryotic organisms revealed that as in *T. thermophila*, the ends of chromosomes were constituted by a block of tandemly repeated simple sequences, whose number varied depending on the organism. Furthermore, the composition of telomeric DNA was asymmetric, with the DNA strand running in the 5′ to 3′ direction rich of guanine and longer than the complementary strand [5].

The fact that telomeres from the same organism could be of different lengths suggested that telomeric DNA was not templated by the parental chromosome. This hypothesis was strengthened by Szostak and Blackburn, who ligated *T. thermophila* rDNA telomeres onto both ends of a linear yeast plasmid and introduced it into yeast by transformation [6]. They found that yeast cells maintained the plasmid as a linear molecule. Furthermore, cells were capable to add the yeast C_1–3_A telomeric repeats at the tips of *T. thermophila* rDNA telomeres [7], arguing that the structural features required for telomere replication had been conserved during evolution.

The finding that telomere elongation was entirely attributable to an increase in the number of tandemly repeated units led Blackburn and Szostak to propose the existence of a terminal transferase-like activity that was capable to add telomeric repeats onto chromosome ends, and that *T. thermophila* DNA ends, but not random sequence, could be recognized as a substrate by the “DNA addition enzyme” [7]. By using cell-free extracts from *T. thermophila*, Greider and Blackburn first uncovered the existence of a terminal transferase activity capable of adding DNA repeat sequences to the chromosome ends [8]. Purification of this enzyme, named telomerase, allowed them to show that it was a ribonucleoprotein complex, whose RNA and protein components were both essential for its activity [9]. The RNA component was then cloned and it was found to contain a short RNA sequence that could act as a template for the addition of simple repeats [10]. Mutation of this sequence caused the introduction, in vivo, of telomere sequences corresponding to the mutated sequence, thus providing the proof that telomerase used its integral RNA component as the template for the addition of simple repeat units [11]. 

In parallel, the search for *S. cerevisiae* mutants that were impaired in the ability to convert a circular plasmid containing inverted repeats of *T. thermophila* telomeric sequences into a stable linear form allowed Lundblad and Szostak to discover the first gene encoding one of the subunits of the yeast telomerase enzyme [12]. This gene was called *EST1*, because its mutation led to loss of telomeric DNA, giving rise to the so-called “ever shorter telomere” (est) phenotype. *est1* mutants also showed a gradual decline of cell viability, thus providing the first experimental demonstration that loss of telomeric DNA limits cellular proliferation. As the lack of Est1 caused chromosome loss, three additional *EST* genes (*EST2*, *EST3*, and *EST4*) were discovered in a screen for mutants that exhibited increased chromosome instability combined with defects in plasmid linearization [13]. The *TLC1* (telomerase component 1) gene encoding the template RNA of telomerase was identified by Singer and Gottschling in a search for genes that, when overexpressed, counteract the ability of telomeres to silence transcription [14]. Purification of the catalytic subunit of telomerase from a ciliated protozoan allowed to demonstrate that the reverse transcriptase motifs were essential for telomeric DNA synthesis in vivo and in vitro [15]. This discovery was followed by the cloning of human telomerase RNA component (TERC) [16] and the telomerase reverse transcriptase (TERT) [17].

## 2. The DNA Damage Response

**Figure 2:**
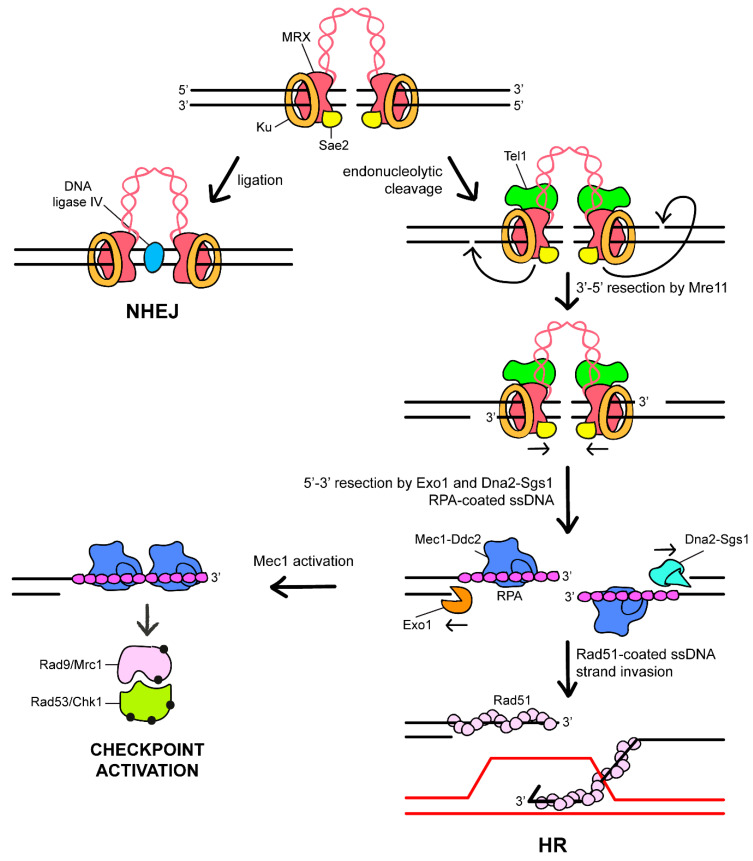
Overview of the DDR at DSBs in *S. cerevisiae*. DSBs can be repaired by non-homologous end-joining (NHEJ) or homologous recombination (HR). MRX-Sae2 and Ku protein complexes are recruited to the DSB. MRX is required to load Tel1. In NHEJ, Ku acts as a hub to recruit downstream NHEJ components, including the DNA ligase IV that catalyzes direct ligation of the DSB ends. If the DSB is not repaired by NHEJ, upon ATP hydrolysis by Rad50, Mre11 together with Sae2 catalyzes an endonucleolytic cleavage of the 5′-terminated strands, followed by bidirectional resection catalyzed by Mre11 in the 3′ to 5′ direction and by Exo1 or Dna2-Sgs1 in the 5′ to 3′ direction. RPA binds to the 3′-ended ssDNA overhangs and is then replaced by Rad51. The Rad51-ssDNA intermediate initiates the homology search, invades the dsDNA, and pairs with the homologous DNA strand. RPA-coated ssDNA recruits the Mec1-Ddc2 complex, which eventually leads to checkpoint activation by activating Rad53 and/or Chk1 through the adaptor Rad9 or Mrc1. Black dots indicate phosphorylation events.

Although telomeric DNA is structurally similar to the end of a DSB, it is intrinsically refractory to repair and does not activate the DNA damage response (DDR) that is, instead, elicited by an intrachromosomal DSB. In eukaryotic cells, the DDR comprises pathways to repair DNA breaks and a mechanism, called DNA damage checkpoint, that inhibits cell-cycle progression until DNA lesions are repaired [18]. The main mechanisms repairing a DSB are non-homologous end-joining (NHEJ) and homologous recombination (HR) (Figure 2). NHEJ catalyzes the direct ligation of the DSB ends and requires the Ku complex that comprises the two Ku70 and Ku80 subunits. This protein complex acts as a hub for the recruitment of downstream NHEJ components, including *S. cerevisiae* Lif1 (human XRCC4), Nej1 (human XLF), and the DNA ligase IV [19]. The presence of Ku at DSBs also protects the DNA ends from degradation by inhibiting the recruitment of the Exo1 nuclease [20,21]. By contrast, HR is a more complex process that uses undamaged homologous DNA as a template to restore the genetic information lost at the break site [22].

The key step in determining which pathway is used to repair a DSB is the initial nucleolytic degradation of the DSB ends. While NHEJ requires little or no DSB end-processing, initiation of HR requires that the 5′-terminated strands of the DSB ends are nucleolytically degraded, in a process called resection, to generate 3′-ended single stranded DNA (ssDNA) [23]. Extended resection of the DSB ends not only commits DSB repair to HR, but it makes also the DNA ends refractory to be ligated by the NHEJ machinery. The resulting 3′-ended ssDNA is first coated by the ssDNA binding complex replication protein A (RPA), which is then replaced by the recombinase Rad51 to form a right-handed helical nucleoprotein filament for homology search and strand invasion (Figure 2). The invading DNA end serves to prime DNA synthesis using the intact homologous DNA sequence as a template, followed by resolution of the resulting DNA structure and ligation [22].

In both yeast and mammals, DSB resection requires the evolutionarily conserved MRX/MRN protein complex that is composed of Mre11, Rad50, and Xrs2 (human MRE11, RAD50, and NBS1) subunits [24] (Table 1). This complex possesses a hetero-hexameric structure, in which Mre11 dimerizes and interacts with both Rad50 and Xrs2/NBS1. While Mre11 exhibits 3′-5′ exonuclease and endonuclease activities [25,26], Rad50 is an ATPase that possesses two antiparallel coiledcoil domains that can dimerize through a Zn-hook motif [24]. During DSB resection, the Sae2 (human CtIP) protein stimulates a latent Mre11 endonuclease activity within the context of the MRX complex to cleave the 5′-terminated strands on either side of the DSB [27]. The resulting nick generates an entry site for Mre11 exonuclease, which degrades back toward the DSB end in a 3′-5′ direction, and for Exo1 and Dna2 nucleases that degrade DNA away from the DSB in a 5′-3′ direction [28,29,30,31,32,33,34,35] (Figure 2). Dna2 processing activity requires the RecQ helicase Sgs1 (human BLM) that unwinds double-stranded DNA (dsDNA) and generates a substrate for Dna2 that cleaves ssDNA overhangs adjoining duplex DNA (Table 1). The ATPase activity of Rad50 drives conformational changes of the complex that modulate its functions. In particular, the Rad50 dimer, when bound to ATP, prevents the access of Mre11 to dsDNA [36,37,38,39], whereas ATP hydrolysis induces a dissociation of the Rad50 nucleotide binding domains and the reposition of Mre11 to one side of Rad50 dimer. This conformational change generates a DNA cutting channel that allows Mre11 to bind dsDNA and to endonucleolytically process it [37,40].

DSB occurrence can elicit activation of a DNA damage checkpoint response, which couples DSB repair with cell-cycle progression [18]. Key checkpoint players include the apical protein kinases Tel1 and Mec1, whose mammalian orthologs are ATM (ataxia telangiectasia mutated) and ATR (ataxia telangiectasia and Rad3-related), respectively (Table 1). Tel1, which was originally identified for its requirement to elongate *S. cerevisiae* telomeres [41], is the kinase involved in sensing and signaling unprocessed or minimally processed DNA DSBs. In both yeast and mammals, recruitment and activation of Tel1/ATM require the MRX/MRN complex [42,43,44,45,46]. Tel1, in turn, once loaded at DSBs by MRX, supports MRX function in a positive feedback loop by promoting/stabilizing its association to the DSB [47].

Upon DSB resection, the replication protein A (RPA) complex binds the ssDNA overhangs and promotes recruitment of Mec1/ATR kinase [48]. Mec1, as well its human ortholog ATR, interacts with Ddc2 (human ATRIP) that helps its recruitment to the DSB ends [49]. Once Mec1/ATR is activated by RPA-coated ssDNA, it phosphorylates and activates the downstream checkpoint kinases Rad53 (human CHK2) and Chk1 (human CHK1), which control two parallel branches of the checkpoint [50]. Signal transduction from apical to downstream checkpoint kinases requires the mediator proteins Rad9 (human 53BP1) and Mrc1 (human Claspin) (Figure 2) (Table 1). In particular, Rad9 allows Rad53 phosphorylation and checkpoint activation in response to DNA damage in the G1 and G2 phases [51,52], whereas Mrc1, which is a component of the replisome, promotes Rad53 activation during S phase [53,54,55,56].

## 3. Telomere Capping and the Consequence of Its Loss

Following the discovery that the ends of chromosomes should possess a unique structure that prevents their fusion, *S. cerevisiae* cells have been used to demonstrate that, when a short array of telomeric DNA repeats was inserted immediately adjacent to an endonuclease-induced DSB, the break was not subjected to fusions by NHEJ because of its failure to recruit the DNA ligase IV [57,58,59]. Furthermore, this DSB was unable to elicit a checkpoint response [57,58], indicating that telomeres exert an “anticheckpoint” activity. Subsequent studies have established that suppression of DNA repair and DNA damage checkpoint at telomeres, referred to as capping, relies on proteins specifically present or enriched at single-stranded and double-stranded telomeric DNA that, in budding yeast, include the protein complexes Cdc13-Stn1-Ten1 (CST), Ku70-Ku80 (Ku), and Rap1-Rif1-Rif2 (Table 1).

### 3.1. The CST Complex

**Figure 3:**
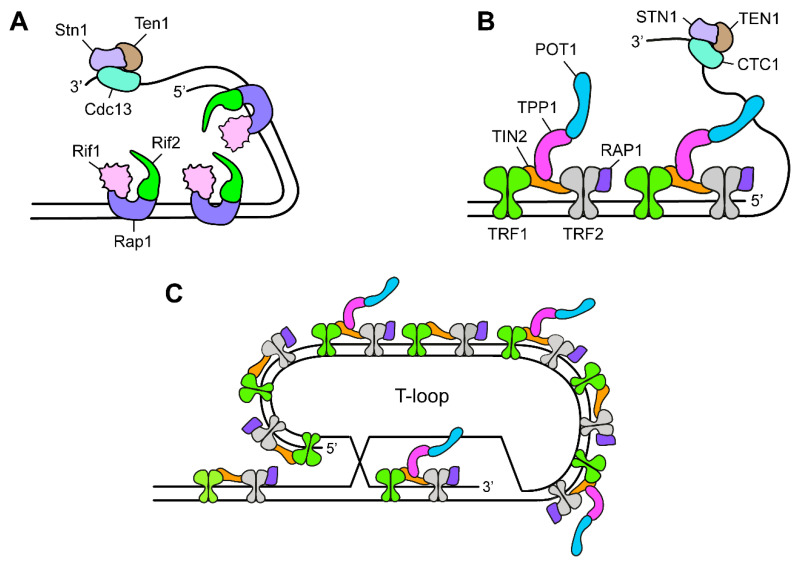
Telomeric structure and capping proteins in yeast and humans. (**A**) Schematic representation of the yeast Rap1-Rif1-Rif2 and CST complexes. CST in yeast is composed of Cdc13, Stn1, and Ten1 proteins. (**B**) Schematic representation of the mammalian shelterin complex, composed of TRF1, TRF2, TIN2, RAP1, TPP1, and POT1 subunits, and the CST complex, composed of CTC1, STN1 and TEN1 subunits. (**C**) The G-rich 3′-ended single stranded overhang bound by the shelterin complex is looped back into the telomeric DNA to form a telomeric loop (T-loop).

The earliest demonstration of the existence of specialized proteins that distinguished the chromosome ends from internal DSBs was the discovery that yeast cells, carrying a temperature-sensitive mutation in the *CDC13* gene, incubated at restrictive temperatures, degraded their telomeres resulting in extensive ssDNA that activates a Rad9/Mec1-dependent checkpoint [60,61]. Cdc13 interacts with Stn1 and Ten1 proteins to form a telomeric ssDNA binding complex called CST (Figure 3A). Both Stn1 and Ten1 support Cdc13 capping activity. In fact, exposure of cells harboring *stn1* or *ten1* conditional alleles to restrictive conditions causes telomere degradation and checkpoint-mediated cell-cycle arrest [62,63,64]. This protein complex is highly conserved and has been identified in ciliates, vertebrates, flies, and plants [65]. 

Subsequent studies have shown that the CST complex has structural similarities with the single-strand DNA binding complex RPA [66], but with a preferential binding to telomeric G-rich ssDNA overhangs. As Mec1 recognizes RPA-coated ssDNA, CST has been proposed to inhibit Mec1 activation by blocking RPA from gaining access to the telomeric single-stranded overhangs, thus limiting Mec1 loading onto DNA and, therefore, its activation [67].

The use of an inducible degron allele of Cdc13 allowed to demonstrate that the CST complex exerts its capping function during late S and G2/M phases of the cell cycle, but not in G1 or early S [68,69]. Interestingly, passage through S phase in a temperature sensitive *cdc13* mutant causes generation of Exo1-dependent ssDNA and unstable chromosomes that are then the source for additional chromosome instability events [70]. This genome instability has been shown to be due to defects in telomere replication, suggesting that the Cdc13 capping function relies on its involvement in supporting replication of telomeric DNA. Consistent with this hypothesis, Cdc13 and Stn1 physically interact with the polα-primase complex and promote its recruitment to the telomeric DNA to fill-in the C-strand [71,72,73]. As telomere binding proteins can represent intrinsic obstacles for replication fork progression [74,75], the role of CST in supporting polα-primase activity could facilitate repriming on the lagging strand to compensate for fork stalling that inherently occurs during telomere replication.

The CST complex in mammals is comprised of CTC1, STN1, and TEN1 subunits [65] (Figure 3B). When CTC1 is disrupted, the G-rich 3′-ended overhangs elongate, while the C-strands decrease in length due to a deficiency in C-strand fill-in synthesis [76,77,78]. The role of CST as a repriming complex at telomeres was also proposed for the mammalian CST [79,80], which appears to have extratelomeric functions in DNA replication and fork restart in the presence of replication stress [81]. 

### 3.2. The Ku Complex

In the G1 phase of the cell cycle, telomere capping relies on the Ku complex, which is an evolutionarily conserved heterodimer composed of Ku70 and Ku80 (human KU70 and KU80) subunits. In *S. cerevisiae*, Ku restrains degradation of telomeric DNA and checkpoint activation [82,83,84]. The increased ssDNA and the activated checkpoint response in cells lacking any Ku subunit at elevated temperatures can be suppressed by deletion of *EXO1* [83], suggesting that Ku represses Exo1 activity at telomeres. Consistent with this hypothesis, the phenotypes caused by Ku dysfunction can be suppressed also by the overexpression of either Est2 reverse transcriptase or TLC1 RNA template, whose high levels appear to stabilize telomeres by enhancing their resistance to degradation by Exo1 [85,86]. The Ku complex is constitutively present also at human telomeres, where it protects telomeric DNA from degradation and HR, although this repression in Ku-deficient mouse cells involves also RAP1 and POT1 proteins that belong to the shelterin complex (see next paragraph) [87,88,89].

### 3.3. The Rap1-Rif1-Rif2 Complex

In *S. cerevisiae*, the other protein complex with capping function is composed of Rap1, Rif1, and Rif2 proteins, with Rap1 binding directly double-stranded telomeric DNA (Figure 3A). These proteins also negatively regulate telomere length by controlling different pathways [90,91]. In *S. cerevisiae*, Rap1 and Rif2, and to a much lesser extent Rif1, repress telomere-telomere fusions by NHEJ, telomere degradation, and checkpoint activation [58,68,84,92]. Rif2 also inhibits activation of Tel1, which is known to promote telomerase-mediated telomere elongation [93,94]. By contrast, Rif1, but not Rif2, is important to support viability in cells where Cdc13 is dysfunctional [95]. Thus, Rap1 and its interactors Rif1 and Rif2 have capping activities, with Rif1 and Rif2 making specific and separable contributions to this capping.

In mammals, the capping properties of the Rap1-Rif1-Rif2 complex are functionally recapitulated by a protein complex, called shelterin, which is composed of TRF1, TRF2, RAP1, TIN2, TPP1, and POT1 subunits [96] (Figure 3B). TRF1 and TRF2 bind to TIN2. TIN2 also binds to TPP1, which in turn binds to POT1, whereas RAP1 binds TRF2. RAP1 is the only shelterin subunit possessing a limited conservation with its yeast ortholog Rap1, although yeast Rap1 binds directly telomeric DNA, whereas the association of human RAP1 with DNA is mediated by TRF2.

The shelterin complex uses a variety of strategies and different subunits to block the DDR activities at telomeres. In particular, TRF2 prevents ATM activation, whereas POT1 is used to repress ATR signaling activity [97]. As POT1 binds ssDNA, the proposed model is that POT1 blocks RPA from gaining access to the telomeric single-stranded overhang, thereby limiting ATR activation. TRF2 is also the main inhibitor of classical NHEJ (c-NHEJ), a pathway responsible for the generation of end-to-end fusions and dicentric chromosomes that can result in breakage-fusion-bridge cycles and genome instability [98]. Artificial tethering of TRF2 next to a DSB impedes its repair and elicits prolonged DDR activation, suggesting that TRF2 is both necessary and sufficient to suppress c-NHEJ [59]. The main mechanism through which TRF2 exerts this inhibitory function is based on formation of T-loops, which are large lariat structures that are generated through strand invasion of the long 3′-ended overhang into the double-stranded telomeric DNA [99,100] (Figure 3C). TRF2 was also found to limit ATM signaling directly by inhibiting the kinase activity itself [101].

The shelterin complex also represses an end-joining pathway, called alternative NHEJ (alt-NHEJ), which is mediated by poly(ADP-ribose) polymerase 1 (PARP1), DNA ligase III, and the error-prone translesion DNA polymerase θ [102]. While c-NHEJ leads to minimal sequence alterations, alt-NHEJ causes extensive deletions and insertions at the repair junction. Furthermore, it is responsible for telomere fusions in senescent cultured cells and in human cancers, suggesting that it might be involved in the processing of dysfunctional telomeres in the early stages of tumorigenesis [103]. Activation of alt-NHEJ can be observed only when all the shelterin subunits are completely depleted in mouse cells that lack the Ku complex [104], suggesting that repression of alt-NHEJ at telomeres involves multiple proteins that act in a redundant manner. 

Although there are significant differences in the sequences and proteins at yeast and human telomeres, human TRF1 and TRF2 share with *S. cerevisiae* Rap1 the DNA binding domain with two Myb-like folds. However, while TRF1 and TRF2 contain a single Myb-like domain, *S. cerevisiae* Rap1 binds a recognition sequence through two tandem Myb-like domains [105,106,107]. In yeast Rap1, a wrapping loop, immediately after the C-terminal Myb-like domain, folds back and locks Rap1 around DNA by interacting with the Myb domain located at the N-terminus. The transient opening of the wrapping loop destabilizes this clamped structure and allows Rap1 binding to DNA through a single Myb-like domain [108,109]. These different Rap1 DNA binding modes were shown to influence Rap1 ability to interact with Rif2 in vivo [110]. In fact, Rap1 mutant variants that increase or decrease Myb affinity to DNA, as well as mutational impairments of the wrapping loop clamping, showed that binding of both Myb-like domains to DNA results in Rap1-DNA complexes that act primarily through Rif2 to control MRX functions at telomeres. By contrast, the transition to a binding mode where a single Myb-like domain is bound to DNA leads to Rap1-DNA complexes that inhibit MRX function at telomeres in a Rif2-independent manner [110].

In any case, the ability of Rif2 to counteract Tel1 activation, NHEJ, and nucleolytic degradation of telomeric DNA appears to rely on inhibition of MRX activity at telomeres [58,68,84]. Because Rif2 interacts with Xrs2 C-terminus within the same region as Tel1 [94], Rif2 was initially proposed to inhibit MRX association/persistence to telomeric DNA ends by competing with Tel1 for Xrs2 binding, therefore antagonizing Tel1-mediated stabilization of MRX association with DNA ends. However, the finding that Rif2 interacts in vitro with Rad50 and can inhibit MRX-dependent stimulation of Tel1 kinase activity independently of Xrs2 [47,111], suggests that Rif2 can act directly on Rad50 to control MRX activity at telomeres. Interestingly, Rif2 was shown to stimulate ATP hydrolysis by Rad50 in an Xrs2-independent manner [47,111]. As MRX binding to DNA, as well as its ability to stimulate Tel1 activation and NHEJ requires that Rad50 is bound to ATP [36,39,112,113,114], Rif2 can inhibit all these MRX functions by discharging the MRX ATP-bound conformation through stimulation of Rad50 ATPase.

Sae2 is required to stimulate Mre11 endonuclease activity [27]. Interestingly, Rif2 was recently shown to inhibit Mre11 endonuclease activity within the context of the MRX complex [115,116]. Genetic and structural modelling approaches identified K6, N18, K81, and I93 residues on Rad50 as being important to support Rad50-Rif2 interaction and Rif2-mediated inhibition of Mre11 endonuclease [115,116,117]. Notably, two of these amino acids belong to a cluster of residues found to be mutated in the meiosis-defective *rad50-S* alleles, which specifically impair Mre11 endonuclease activity by abrogating Rad50-Sae2 interaction and, therefore, Sae2-mediated stimulation of Mre11 nuclease [118]. Altogether these findings suggest that Sae2 and Rif2 interaction interfaces can partially overlap on Rad50, raising the possibility that Rif2 can inhibit Mre11 endonuclease by competing with Sae2 for Rad50 binding and, therefore, by limiting Sae2-mediated MRX stimulation.

**Figure 4:**
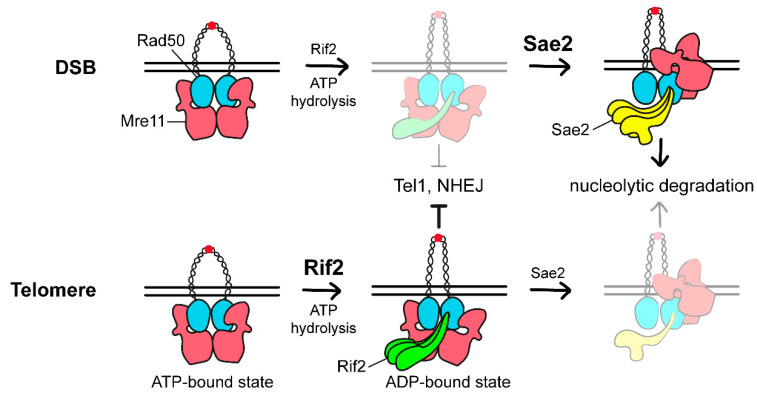
Model of regulation of MRX activity at DSBs and telomeres. In the ATP-bound state, the dsDNA is inaccessible to Mre11. Upon ATP hydrolysis by Rad50, the two Rad50 coiled coils zip up and Mre11 moves to the side of Rad50 dimer where it can act as endonuclease. At the DSB, the excess of Sae2 compared to Rif2 leads to Sae2 binding to the Rad50-Mre11 interface. This interaction stabilizes Mre11-Rad50 in a conformation that is proficient to cleave DNA. At telomeres, the excess of Rif2 compared to Sae2 antagonizes Sae2 binding to Rad50 and stabilizes a post hydrolysis ADP-bound state that is not competent for NHEJ, Tel1 activation, and DNA cleavage. Xrs2 is not represented.

Although Rif2 can be detectable also at DSBs [47], it is much more abundant at telomeres compared to Sae2, which in turn is avidly bound to DSBs. The different enrichment of these two proteins at DSBs versus telomeres provides the rationale to explain why Rif2 inhibits MRX-mediated resection preferentially at telomeres. In fact, at DSBs Rif2 only represses Tel1 activation and NHEJ by discharging the MRX ATP-bound state [47], whereas it fails to inhibit resection possibly because it is not enough to antagonize Sae2 binding to Rad50 and, therefore, the conversion of MRX into an endonuclease active complex (Figure 4). 

Rif2 functions in inhibiting Mre11 endonuclease, Tel1 activation, and NHEJ depend on a small Rif2 region of as little as 34–40 amino acids, called MIN (MRN-INhibitor) [111,115,117]. The MIN motif, which belongs to the Rif2 BAT (Blocks Addition of Telomeres) motif (residues 1–60), previously shown to be involved in the negative regulation of telomere length [119], mediates also Rif2 binding to Rad50 and stimulation of ATPase activity. The finding that Rad50 K6 and K81 residues are evolutionarily conserved (human K6 and R83) raises the possibility that an analogous mechanism might be deployed in other eukaryotes.

## 4. Consequences of Telomere Shortening

Telomere attrition can be caused not only by disrupting the interaction between the telomeric DNA and the proteins that specifically bind to it, but also by eliminating the telomerase activity. In 1961, Leonard Hayflick discovered that human fibroblasts derived from fetuses possessed finite replicative potential of 50–60 doublings. Then, they entered a non-dividing state called replicative senescence or the “Hayflick limit” [120]. Based on the finding that DNA polymerases replicate DNA only in the 5′ to 3′ direction and need a primer to initiate DNA synthesis [121], Alexei Olovnikov, in its theory of marginotomy, predicted that the conventional DNA replication machinery cannot replicate completely the chromosome ends that would incur a loss of DNA from the lagging strand, leading to progressive chromosome shortening. He proposed that this shortening can account for the limitation of doubling potential of normal somatic cells [122].

*S. cerevisiae* cells possess a constitutively active telomerase, but a senescence phenotype can be induced following telomerase removal. The first experimental demonstration that loss of telomeric DNA limits cellular proliferation comes from the discovery that yeast cells lacking the Est1 subunit of telomerase showed a gradual arrest of doubling [12]. Similarly, human cultured primary cells shorten their telomeres as a function of serial passage during ageing [123]. Furthermore, re-expression of the catalytic subunit of telomerase can extend the lifespan of telomerase-negative human cells [124]. 

One hypothesis to explain the decrease in growth capacity was that chromosome erosion leads to genetic instability that causes cell death. Indeed, it was shown that yeast cells deleted for the *EST1* gene increase the frequency of gross chromosomal rearrangements involving terminal, but not internal, deletions [125]. However, these events can be detectable only after a significant loss of growth potential, suggesting that chromosome instability was not the major determinant of the senescent phenotype. Rather, loss of growth in these yeast cells correlated with an arrest of the cell cycle in the G2 phase and activation of Rad53 checkpoint kinase [126,127]. This response was dependent on genes involved in the DNA damage checkpoint, indicating that checkpoint activation at critically short telomeres is the trigger of replicative cellular senescence. Similarly, human cells, in which the telomerase activity is downregulated, undergo telomere shortening and prolonged DDR signaling, which results in the formation of telomere-induced DNA damage foci (TIFs) that colocalize with DNA repair and DNA damage checkpoint proteins [128,129]. Furthermore, overexpression of the shelterin subunit TRF2 can delay senescence onset [130], arguing that checkpoint activation at telomeres can be elicited not only as a consequence of telomerase inactivation but also of insufficient amounts of shelterin bound at telomeres.

**Figure 5:**
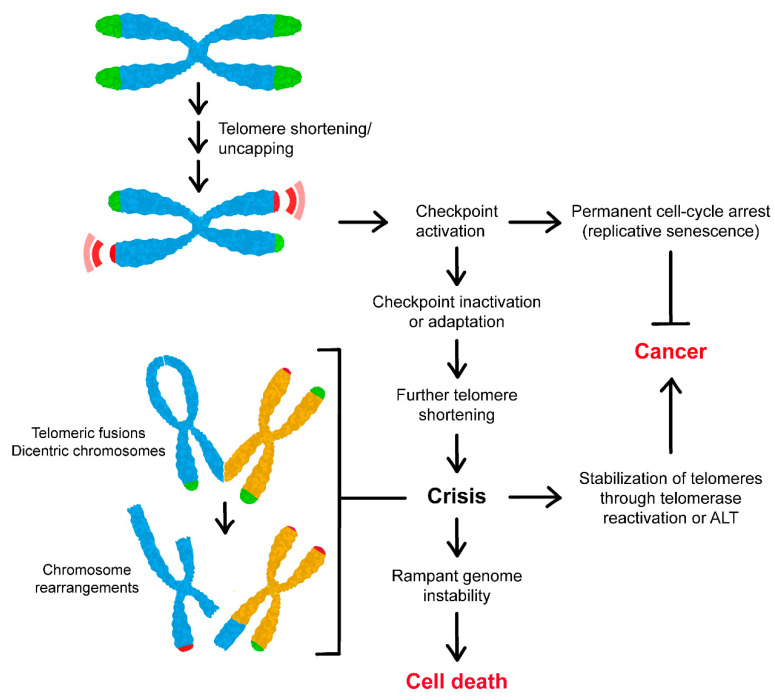
Telomere attrition in cancer. Telomere attrition due to either deficiency in capping proteins or loss of telomeric DNA induces a DNA damage checkpoint response that leads to a permanent cell-cycle arrest and entry into a non-dividing state called replicative senescence, which provides a potent anticancer barrier. However, checkpoint inactivation through mutations or adaptation allows cells to bypass senescence, reaching a second proliferative state, known as telomere crisis, during which critically short telomeres become vulnerable to end-to-end fusions, forming dicentric chromosomes and resulting in deletions, amplifications, and translocations. This rampant genomic instability leads to death of most cells, but the few cells that re-stabilize telomeres by re-activating telomerase or inducing ALT can survive and proliferate indefinitely, thus promoting malignant transformation.

Altogether, these findings lead to a model whereby as telomeres shorten, they become progressively unable to bind telomere-capping proteins, thus resembling one-ended DSBs (Figure 5). This change in the protected status leads to activation of a checkpoint that is similar to that triggered by intrachromosomal DSBs and that permanently arrests cells in replicative senescence. It has been proposed that such short telomeres are not subjected to fusion events, possibly because they retain sufficient shelterin complex to inhibit them [131]. However, inactivation of the checkpoint allows these cells to bypass the senescent state, reaching a second proliferative barrier, known as telomere crisis, in which critically short telomeres become vulnerable to fusion events and formation of dicentric chromosomes [132,133]. Although most cells die, the few cells that re-elongate their telomeres proliferate indefinitely. Telomere shortening and activation of the DNA damage checkpoint occur also in ageing post-mitotic cells, including cardiomyocytes, adipocytes, neurons, osteocytes, and osteoblasts [134], suggesting that cellular senescence contributes to organismal ageing. 

Telomerase-negative *S. cerevisiae* cells, where a single telomere was engineered to be reduced in length without affecting the integrity of its tip, accelerate the onset of senescence [135,136]. This signaling telomere is bound by RPA, Rad52, Ddc2, and Mec1 repair and checkpoint proteins, indicating that a single very short telomere is sufficient to induce checkpoint activation and replicative senescence in yeast. Similarly, the presence of few very short telomeres in mouse cells is sufficient to trigger replicative senescence [137], indicating that is not the average but rather the presence of one or few critically short telomeres that causes checkpoint activation and senescence entry. Importantly, Mec1 is required to induce a senescent state in the presence of a critically short telomere [135], suggesting that ssDNA is the primary signal triggering senescence. Consistent with this hypothesis, 5′-3′ resection is stimulated at short telomeres that expose subtelomeric ssDNA [138]. Furthermore, the lack of telomere-processing proteins, such as MRX, delays senescence, while the lack of MRX inhibitors, such as Rif2, anticipates it [139]. 

The absence of Tel1 also delays senescence [126,135,139,140]. As Tel1 promotes ssDNA generation at both DSBs and telomeres [141], the delayed senescence in Tel1-deficient telomerase-negative cells can be due to a reduced amount of telomeric ssDNA. By studying the senescence phenotype of telomerase-deficient cells lacking Tel1 or expressing the hyperactive Tel1-hy184 mutant variant, which has been identified because of its ability to compensate for the lack of Mec1 function [142], it was shown that Tel1-hy184 anticipates senescence, while the lack of Tel1 or of its kinase activity delays it [143]. Neither Tel1-hy184 nor Tel1 kinase defective variant affects the generation of ssDNA at telomeres, suggesting that Tel1 function in promoting senescence is not directly linked to ssDNA generation. The finding that the anticipated senescence triggered by Tel1-hy184 completely depends on Rad9 and only partially on Mec1 suggests that Tel1 promotes senescence mainly by directly signaling the presence of dysfunctional telomeres to a Rad9-dependent checkpoint. These results suggest that, as telomeres shorten in the absence of telomerase and the negative control exerted by Rif2 on MRX-Tel1 activity declines, MRX recruits Tel1 at telomeres that can directly signal to the checkpoint machinery.

## 5. Escape from Telomere-Induced Replicative Senescence

Replicative senescence elicited by activation of the checkpoint response is a state of stable, terminal cell-cycle arrest that acts as a barrier against tumorigenesis. Importantly, telomere attrition in ageing telomerase-deficient mice lacking the checkpoint protein p53 was reported to cause epithelial cancers by a process of breakage-fusion-bridge [144], indicating that the status of the checkpoint response dictates whether the short telomeres promote or suppress cancer. 

The checkpoint response can be overcome either through mutational inactivation of its components or through adaptation, which is a phenomenon originally described in yeast as the ability of cells to overcome a sustained checkpoint arrest despite the presence of unrepaired DNA damage. The ability of cells to adapt to altered telomere length or structure has been first observed in *S. cerevisiae* by Sandell and Zakian, who discovered that, after elimination of a single telomere and checkpoint activation, many cells were capable to resume cell-cycle progression without having repaired the damaged chromosome [3]. In budding yeast, adaptation has been observed also in response to a single unrepairable DSB [145,146,147], where it requires the polo kinase Cdc5 [145], the phosphatase Ptc2 [148], the regulatory subunits Ckb1 and Ckb2 of casein kinase II (CKII) [145], and the recombination proteins Tid1 and Srs2 [149]. During adaptation, Rad53 checkpoint kinase is inactivated [147], thus allowing cells to resume cell-cycle progression to get an opportunity to repair DNA damage in other cell-cycle phases [150,151]. Mechanisms to abrogate a prolonged checkpoint arrest were also reported in *Xenopus laevis* and human cells and a similar genetic requirement suggests a common evolutionary origin [152,153].

Budding yeast cells are capable to adapt to the checkpoint that is elicited not only in response to loss of telomerase but also to capping defects. In fact, downregulation of the checkpoint response can allow *cdc13* mutant cells to adapt to the presence of uncapped telomeres and resume cell-cycle progression [145,154]. In these cells, uncapped telomeres persist throughout the cell cycle and are bound by DNA repair proteins, indicating that the resuming of cell-cycle progression is not due to repair of damaged telomeres but to abrogation of the checkpoint response. Proteins, such as Tid1, Ptc2, and Cdc5, known to be required for adaptation to a single unrepaired DSB, are required to allow adaptation of *cdc13* mutant cells [154], arguing that adaptation to a DSB or to capping defects occurs by a similar mechanism. 

By tracking individual cell lineages over time using a microfluidic-based approach coupled to single-cell imaging, it was found that adaptation-deficient cells have a higher mutation rate than adaptation-proficient cells [155], indicating that adaptation contributes to genome instability. This genome instability can be due to end-to-end telomeric fusions to form dicentric chromosomes and subsequent breakage-fusion-bridge cycles [156]. The increased genome instability in adapted cells might be due also to the use of mutagenic repair pathways, such as NHEJ in G1 or microhomology-mediated end-joining, that were not utilized during a checkpoint-mediated cell-cycle arrest [150].

The rampant chromosome instability experienced by adapted cells has important implications for understanding the early steps of tumorigenesis, during which precancerous cells undergo a phase of high genome instability [155]. Although cells undergoing checkpoint adaptation almost die in the subsequent cell cycles due to high levels of genetic instability, cells that overcome this crisis by re-stabilizing critically shortened telomeres can proliferate indefinitely. Consistent with this model, ectopic expression of TERT in combination with two oncogenes was shown to promote malignant transformation of primary human cells [157]. Thus, the chain of events of telomeric alterations, checkpoint activation, and adaptation is a major mechanism that enables malignant transformation in cells lacking telomerase activity (Figure 5). 

## 6. Telomerase-Independent Re-Stabilization of Telomere Length

Although reactivation of telomerase is the most common telomere maintenance mechanism in cancer, cancer cells can use telomerase-independent recombination-based mechanisms, called alternative lengthening of telomeres (ALT), to re-elongate their telomeres. ALT was discovered by Lundblad and Blackburn, who found that yeast cells lacking telomerase can evade the senescence state by re-elongating their telomeres through the use of recombination [158]. Cells that restabilize telomeres by activating such mechanisms have been called post-senescence survivors. Based on telomere organization and genetic requirements, two types of survivors can be described [158,159,160], although other mechanisms can contribute to stabilize telomeres in the absence of telomerase [161,162,163,164]. *S. cerevisiae* telomeres are comprised of ∼300 bp of double-stranded TG_1–3_/C_1–3_A repeats with a 3′-ended 8–15 nucleotides overhang [165,166]. Internal to the TG_1–3_/C_1–3_A tracts are repetitive DNA elements, called X and Y’. Type I survivors arise through amplification of the subtelomeric Y’ sequences that may stem from non-reciprocal translocations or integration of extra-chromosomal Y’ circles into the short telomeres. By contrast, type II survivors harbor long and heterogeneous telomeric repeat tracts with no rearrangement of Y’ elements. Furthermore, type I requires Rad51, whereas type II relies on Rad50, Rad59, and Sgs1 [160,167,168,169,170]. Both types instead depend on the recombination protein Rad52 and the nonessential DNA polymerase δ subunit Pol32. The requirement for Pol32 suggests that break-induced replication (BIR), which is a mechanism used to repair one-ended DSBs through strand invasion into a homologous donor sequence, can be involved [171]. However, a recent analysis of ultra-long sequencing of chromosome ends has revealed that survivors contain DNA sequences that can be attributed to both type I and II [172]. Based on this finding, it was proposed a unified pathway that comprises two sequential steps: formation of precursors by Rad51-mediated strand invasion, followed by maturation into survivors via a Rad59-dependent pathway. In any case, reactivation of telomerase in such cells leads to reversal of the senescence phenotype and restores a telomerase-mediated mode of telomere elongation [173,174], indicating that telomerase represses recombination at telomeres.

The absence of telomerase results in replicative senescence also in *S. pombe* cells that can escape it by maintaining telomeres in a telomerase-independent mode [175]. However, most survivors are formed by circularization of each individual chromosome, possibly because loss of protection due to telomere shortening allows end-to-end fusions to occur [175,176,177]. Interestingly, a new type of survivor, termed HAATI (heterochromatin amplification-mediated and telomerase-independent), that relies on the presence of non-telomeric heterochromatin, has been described in *S. pombe* [178].

ALT mechanisms that depend on homologous recombination can be observed also in human telomerase-negative immortalized cell lines and in 10–15% of human cancers, which use them to re-elongate their telomeres and gain unlimited proliferative potential [179,180]. A plasmid tag inserted into a single telomere in mouse or human ALT cells was found to be copied to other telomeres or duplicated in its original location without the involvement of other telomeres [181,182,183]. This finding suggests that a telomere can use itself or a telomere on a sister chromatid or on another chromosome as a copy template by BIR. Interestingly, the BIR mechanism has been proposed to be responsible also for the generation of type II survivors in yeast [171]. 

The cause of the triggering of HR-based mechanisms at telomeres remains poorly understood. As stated above, telomeres are intrinsic obstacles for replication fork progression in both yeast [74,75] and mammals [184], because of the presence of telomeric DNA-bound proteins and DNA secondary structures. Interestingly, in yeast, phosphorylation of Rad53 upon inactivation of telomerase or of Cdc13 depends not only on Rad9 but also on Mrc1 [185,186], a checkpoint protein implicated in the response to replication stress [53,56]. Furthermore, Mrc1 was found to be phosphorylated during senescence [186], suggesting that replication stresses occur at short telomeres. Bidimensional gels assessing replication intermediates in telomerase-negative fission and budding yeast cells revealed a severe impairment of telomere replication that correlates with an accumulation of four-branch DNA structures [187,188]. Furthermore, using a reconstituted replication assay, it has been shown that budding yeast Rap1 acts as a roadblock to the replisome and potently inhibits lagging strand replication behind the fork [74]. These data support the idea that the absence of telomerase leads to replication stresses at telomeres that force cells to use recombination to repair damaged telomeres and maintain viability. 

In human cells, commitment to ALT is often associated with loss of ATRX/DAXX chromatin remodeling complex, changes in telomeric chromatin, or formation of RNA:DNA hybrids [189,190]. In particular, loss of ATRX causes decompaction/alterations of telomeric chromatin and increased replication stress [191,192,193,194], suggesting that altered telomeric chromatin can drive ALT by inducing replication stress that generates substrates for the recombination machinery [195]. Fork progression can be hampered also by transcription from subtelomeric and telomeric regions that can generate RNA:DNA hybrids, which are structures formed by the annealing of nascent RNA transcripts to the DNA template strand. The best known RNA species that are transcribed from the subtelomeric region toward the chromosome end are long non-coding RNAs conserved in many species that are called TERRA (telomeric repeat-containing RNAs). Interestingly, telomere shortening in both *S. cerevisiae* and *S. pombe* induces TERRA transcription [196,197,198,199], which leads to the generation of RNA:DNA hybrids and an increased frequency of telomere recombination in the absence of telomerase [200,201,202]. Moreover, inhibition of TERRA transcription decreases DNA replication stress and DNA damage at telomeres, and impairs ALT activity and telomere length maintenance by BIR [203].

## 7. Conclusions

Telomere maintenance, which is essential for chromosome integrity, presents multiple challenges. Since the pioneering work of Muller and McClintock, extensive research has revealed that telomeric DNA is bound by a growing list of proteins that serve to regulate its length and protect it from unwarranted fusion, recombination, and degradation events. These results imply that genomes are not uniformly repairable and that some genomic loci, such as telomeric DNA, resist DNA repair. This irreparability may be the consequence of their functions in ensuring the maintenance of linear chromosomes.

Inactivation of telomerase via genetic manipulation in budding yeast recapitulates the process of telomere shortening and induction of replicative senescence observed in human somatic cells. Furthermore, budding yeast uses strategies to escape senescence that resemble those used by human cells. As telomerase removal in budding yeast can allow detection of early and rare events, this organism can be used to decipher the causes and consequences of replication stress at telomeres, the molecular events leading to induction of recombination and telomere-driven mutagenesis. Therefore, the data obtained with this organism can help investigations in mammalian models to make rapid progress in this field that is important for human health.

As precancerous cells undergo rapid proliferation that leads to telomere shortening, it is also clear that, in certain types of cancer, telomere attrition can be a promoter of tumorigenesis. The role of telomere in malignancy and metastasis has been proved in telomerase-deficient checkpoint-mutated mice, in which telomere attrition promotes the development of epithelial cancers by a process of fusion-bridge breakage that leads to the formation of complex non-reciprocal translocations [144,204]. Furthermore, reactivation of telomerase in tumor cells that have already experienced telomere dysfunction is sufficient to dampen the checkpoint response and quell rampant chromosome instability, enabling full malignant transformation [205]. The increased telomerase activity observed in most cancers has led to the development of several strategies to target TERT. However, as TERT inhibition was found to select activation of ALT pathways in lymphoma [206], combined drugs that suppress telomerase and ALT-pathway could be beneficial to minimize emergence of resistance.

## Figures and Tables

**Table 1:** Major proteins involved in the DNA damage response and telomere capping.

*S. cerevisiae*	*H. sapiens*	Description
Mre11-Rad50-Xrs2	MRE11-RAD50-NBS1	DSB sensor; telomere length regulator
Ku70-Ku80	KU70-KU80	DSB sensor; telomere length regulator
Tel1	ATM	Apical protein kinase; telomere length regulator
Sae2	CtIP	Activator of MRX/MRN endonuclease
Exo1	EXO1	Exonuclease
Sgs1	BLM	DNA helicase
Dna2	DNA2	DNA helicase and nuclease
Mec1-Ddc2	ATR-ATRIP	Apical protein kinase and interacting factor
Rad9	53BP1	Checkpoint adaptor/mediator
Mrc1	Claspin	Replisome component; checkpoint activator
Rad53	CHK2	Downstream protein kinase
Chk1	CHK1	Downstream protein kinase
Cdc13-Stn1-Ten1	CTC1-STN1-TEN1	Telomere binding complex; telomere capping regulator
Rap1-Rif1-Rif2	TRF1-TRF2-RAP1-TIN2-TPP1-POT1	Telomere binding complex; telomere capping and length regulator

## Data Availability

Not applicable.
